# From graph models to intelligent decision-making: a review of spatio-temporal graph neural networks for regional disease risk prediction and etiology mining

**DOI:** 10.3389/fpubh.2026.1830634

**Published:** 2026-07-22

**Authors:** Ye Chen, Xiaoqun Qin, Shouping Chen

**Affiliations:** 1 School of Information Science and Engineering, Hunan International Economics University, Changsha, China; 2 Hunan Nianlun Orthopedic Hospital Group, Changsha, China

**Keywords:** disease risk prediction, epidemic early warning, etiology mining, explainable artificial intelligence, health equity, multi-source data fusion, public health surveillance, spatio-temporal graph neural network

## Abstract

**Background:**

Regional disease risk prediction is a core component of public health early warning systems. Traditional statistical models and machine learning methods have inherent limitations in handling multi-source heterogeneous data fusion, complex spatio-temporal dependency modeling, and interpretable etiology mining, making it difficult to meet the demands of precise and real-time public health decision-making.

**Objective:**

This review systematically examines the methodological advances, application scenarios, and future directions of spatio-temporal graph neural networks (ST-GNNs) and multi-source data fusion techniques in regional disease risk prediction and etiology mining, aiming to provide a bridging reference that connects cutting-edge technologies with practical applications for public health researchers, policymakers, and data scientists.

**Methods:**

Following the PRISMA framework, we systematically searched the Web of Science, PubMed, and IEEE Xplore databases for the period 2023–2026, ultimately including 76 core studies. A four-layer methodological framework encompassing graph construction, fusion strategies, spatio-temporal modeling, and interpretable etiology mining was developed.

**Results:**

Representative works are reviewed from two dimensions: prediction tasks (single-disease prediction, multi-disease collaborative forecasting, long-term extrapolation) and etiology mining (spatial transmission tracing, temporal pattern attribution, multi-factor interaction analysis). Five major technical challenges are identified: dynamic graph structure modeling, cross-modal heterogeneous fusion, trade-off between prediction and interpretability, out-of-distribution generalization, and privacy-preserving federated learning. These are complemented by implementation constraints from public health practice, including data availability, computational efficiency, and policy coordination.

**Conclusion:**

The main contribution of this review is the construction of a unified methodological framework integrating prediction and etiology mining, and a systematic synthesis of the challenges and future pathways at the frontier of technology and practical implementation. ST-GNNs demonstrate significant advantages in improving prediction accuracy and interpretability. Future developments should deeply integrate foundation models and causal inference to build a “prediction–intervention–evaluation” closed-loop system, providing actionable methodological references for building regional disease early warning systems and formulating public health intervention strategies globally, especially in low- and middle-income regions, thereby enhancing public health emergency response capacity and health equity.

## Highlights

1. ST-GNNs integrate multi-source data to improve regional disease prediction accuracy and enable interpretable etiology mining.
2. A unified four-layer framework connects graph construction, data fusion, spatio-temporal modeling, and causal inference for public health.
3. Key challenges include dynamic graph adaptation, cross-modal fusion, out-of-distribution generalization, and privacy-preserving learning.
4. Public health constraints—data scarcity, real-time response, health equity—are mapped to technical barriers.
5. Future directions emphasize foundation models, closed-loop decision optimization, and mandatory fairness auditing for global health equity.

## 1 Introduction

Accurate prediction of regional disease risk and the mining of potential causative factors have long been core objectives of public health early warning systems. From traditional influenza surveillance to outbreak warnings of emerging infectious diseases, from geographical studies of chronic diseases to spatio-temporal modeling of vector-borne diseases, researchers have consistently sought to answer two fundamental questions: Where and when will a disease occur? Why does it occur? These questions correspond to prediction tasks and etiology mining tasks, respectively, and they complement each other—accurate prediction requires understanding the causes, while validation of causes relies on prediction.

The cross-regional spread of infectious diseases such as COVID-19 and dengue fever has placed urgent demands on the spatio-temporal prediction capabilities of public health systems. However, in real-world scenarios, data are scattered across multiple sectors (e.g., disease control, meteorology, telecommunications), and transmission mechanisms are dynamically influenced by factors such as population mobility, environmental changes, and policy interventions, making it difficult for traditional methods to support efficient intervention decisions. Traditional disease prediction methods have evolved from statistical models (e.g., ARIMA, SEIR) to classical machine learning approaches (e.g., random forest, support vector machines). While these methods have achieved good results in specific contexts, their limitations are increasingly evident: statistical models struggle to capture complex nonlinear relationships, and traditional machine learning methods often overlook the inherent spatial structure and temporal dependencies within the data. More importantly, these methods typically treat prediction and etiology mining separately, making it difficult to provide interpretable pathogenic mechanisms alongside accurate predictions.

In recent years, the emergence of spatio-temporal graph neural networks (ST-GNNs) has opened new possibilities for overcoming these limitations. Graph neural networks are naturally suited for modeling spatial structures in disease transmission (e.g., inter-regional population flow networks, geographical adjacency relationships), while temporal modeling capabilities (e.g., gated recurrent units, temporal convolutional networks) capture the time-varying dynamics of diseases. When combined with multi-source data fusion techniques, ST-GNNs can integrate multimodal data—meteorological, population mobility, socioeconomic, healthcare resources—within a unified framework, both improving prediction accuracy and revealing key drivers of disease transmission through interpretable components such as attention mechanisms.

### 1.1 Differences from existing reviews

Existing reviews either focus on general applications of graph neural networks in healthcare, emphasize specific epidemic prediction tasks, or address general frameworks for multimodal fusion. Few studies have specifically targeted the scenario of “regional disease risk prediction” by treating “prediction” and “etiology mining” as a unified problem and incorporating a causal inference perspective into methodological analysis. This review not only synthesizes ST-GNN technical advances but also focuses on implementation bottlenecks and solutions in public health practice, providing a reference for technology selection and application for public health researchers and policymakers. The innovations of this paper are: (1) systematically reviewing data fusion strategies and spatio-temporal modeling methods in ST-GNN-based disease risk prediction, with a methodological framework as the main thread; (2) elevating etiology mining to a position equal to prediction, reviewing the application progress of explainable methods and causal inference techniques in this field; (3) based on the latest research (2023–2026), distilling current challenges and future directions, with particular attention to cutting-edge topics such as dynamic graph learning, foundation model integration, causal inference, and health equity.

The structure of this paper is as follows: Section 2 introduces the retrieval methods and basic concepts; Section 3 presents a four-layer methodological framework integrating prediction and etiology mining, elaborating its public health adaptability; Section 4 reviews ST-GNN applications in disease prediction tasks by category; Section 5 focuses on explainability methods and causal inference for etiology mining; Section 6 systematically discusses technical challenges and public health implementation constraints, echoing the adaptability issues raised in Section 3; Section 7 outlines future directions, with special attention to the deep integration of technology and public health action; Section 8 concludes the paper, reaffirming the contribution to global health equity.

## 2 Retrieval methods and basic concepts

### 2.1 PRISMA literature search process

To systematically review research progress on ST-GNNs in regional disease risk prediction from 2023 to 2026, this study followed the PRISMA (Preferred Reporting Items for Systematic Reviews and Meta-Analyses) framework for literature search and screening.

Databases: Web of Science Core Collection, PubMed, IEEE Xplore.
Search period: January 1, 2023 – March 14, 2026.
Search query: (“spatio-temporal graph neural network” OR “ST-GNN” OR “graph neural network” OR “GNN”) AND (“disease prediction” OR “epidemic forecasting” OR “infectious disease” OR “public health surveillance”) AND (“multimodal” OR “multi-source” OR “data fusion”).

#### 2.1.1 Inclusion criteria

(1) Published in peer-reviewed journals or conferences; (2) The research subject is a regional disease (not at the individual level); (3) Methods include graph neural networks and spatio-temporal modeling; (4) Explicitly involve multi-source data fusion; (5) Include prediction or etiology mining tasks.

#### 2.1.2 Exclusion criteria

(1) Use only traditional statistical or machine learning methods; (2) Focus only on individual electronic health records without spatial dimension; (3) Non-English literature; (4) Reviews, comments, editorials, etc., that are not original research; (5) Fail quality assessment (e.g., unclear methods, incomplete results).

#### 2.1.3 Screening process

**Figure 1:**
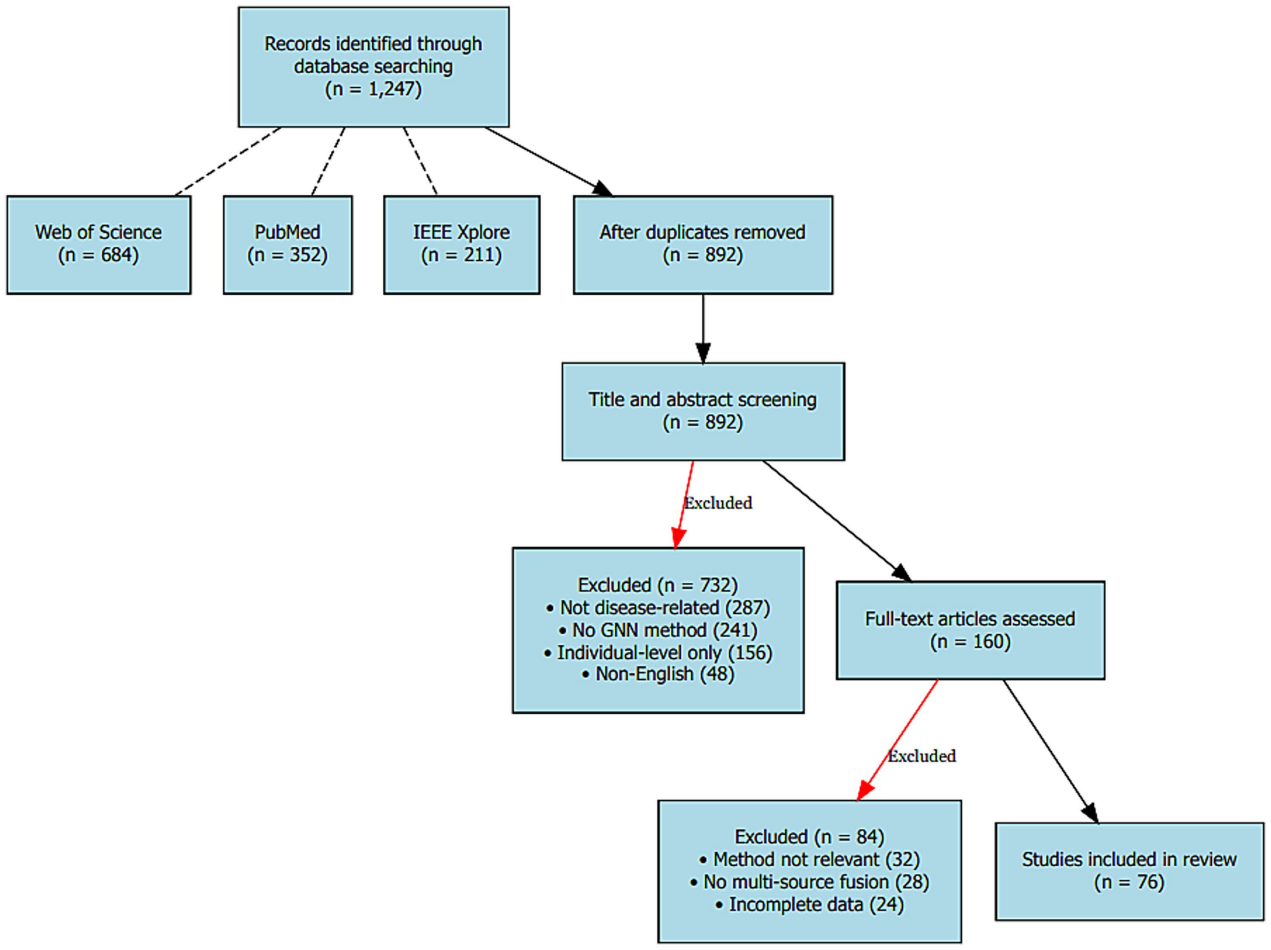
PRISMA flow diagram of literature selection process.

The initial search yielded 1,247 records, reduced to 892 after removing duplicates. After title and abstract screening, 732 records were excluded, leaving 160 for full-text assessment. Full-text evaluation excluded 84 studies (32 with irrelevant methods, 28 without multi-source fusion, 24 with incomplete data), resulting in 76 core studies included in the review. The PRISMA flow diagram is shown in Figure 1.

### 2.2 Core ideas of spatio-temporal graph neural networks

Spatio-temporal graph neural networks extend graph neural networks to spatio-temporal data. The core idea is to abstract the study region into a graph structure, where nodes represent geographical units (e.g., cities, districts/counties) and edges represent spatial relationships between units (adjacency, flow, similarity). Each node is associated with a time-varying feature sequence (e.g., historical case counts, meteorological indicators). The model captures spatial dependencies through graph convolution operations and temporal dependencies through temporal modules, ultimately outputting predictions for future time steps or representations of potential risk.

Mathematically, a spatio-temporal graph neural network layer can be formalized as:

$$H^{\left(l+1\right)}=\sigma (\text{Temporal}(\mathrm{GCN}(H^{\left(l\right)},A)))$$

Where $H^{(l)}$ is the node representation at the $l$-th layer, $A$ is the adjacency matrix, GCN denotes graph convolution operation, Temporal denotes the temporal modeling module (e.g., LSTM, TCN, attention mechanism), and $\sigma$ is the activation function.

Based on the implementation of spatial graph convolution, existing methods can be classified into three categories: (1) Spectral methods: based on spectral decomposition of the graph Laplacian matrix, e.g., Chebyshev polynomial approximation; (2) Spatial methods: direct neighborhood aggregation on the graph, e.g., graph attention networks (GAT); (3) Diffusion convolution methods: define graph convolution based on random walks, e.g., DCRNN. Temporal modeling modules mainly include three architectures: recurrent neural networks (RNN/GRU/LSTM), temporal convolutional networks (TCN), and self-attention mechanisms (Transformer).

### 2.3 Classification and strategies of multi-source data fusion

Regional disease prediction involves an increasingly diverse range of data sources, which can be broadly grouped into four categories: (1) Epidemiological data: historical case counts, incidence rates, mortality, etc.; (2) Environmental and meteorological data: temperature, precipitation, humidity, air quality, etc.; (3) Demographic and socioeconomic data: population density, age structure, GDP, healthcare resource distribution, etc.; (4) Dynamic behavioral data: population mobility trajectories, mobile phone signaling data, social media trends, etc.

Multi-source data fusion in ST-GNNs mainly adopts three implementation levels:

Early fusion: multi-source features are concatenated at the input layer to form a high-dimensional node feature vector, which is then fed into a unified spatio-temporal graph network. The advantage is simplicity, but it may overlook heterogeneity between modalities.
Intermediate fusion: each modality is encoded separately through sub-networks, and fusion occurs at the hidden layer level. This is currently the mainstream strategy, especially using attention mechanisms to dynamically fuse representations from different modalities.
Late fusion: each modality makes independent predictions, and the results are combined via ensemble learning. Suitable for scenarios where modalities are weakly complementary or where independent interpretability of each modality needs to be preserved.

The review by Vaida and Huang (1) systematically examines multimodal graph neural network fusion strategies in healthcare, noting that intermediate fusion combined with attention mechanisms is an effective means of achieving dynamic feature prioritization. Additionally, multimodal fusion must address data missingness—in clinical settings, data for a certain modality is often incomplete, necessitating the design of robust fusion strategies.

## 3 Methodological framework: technical pathways and public health adaptability

**Figure 2:**
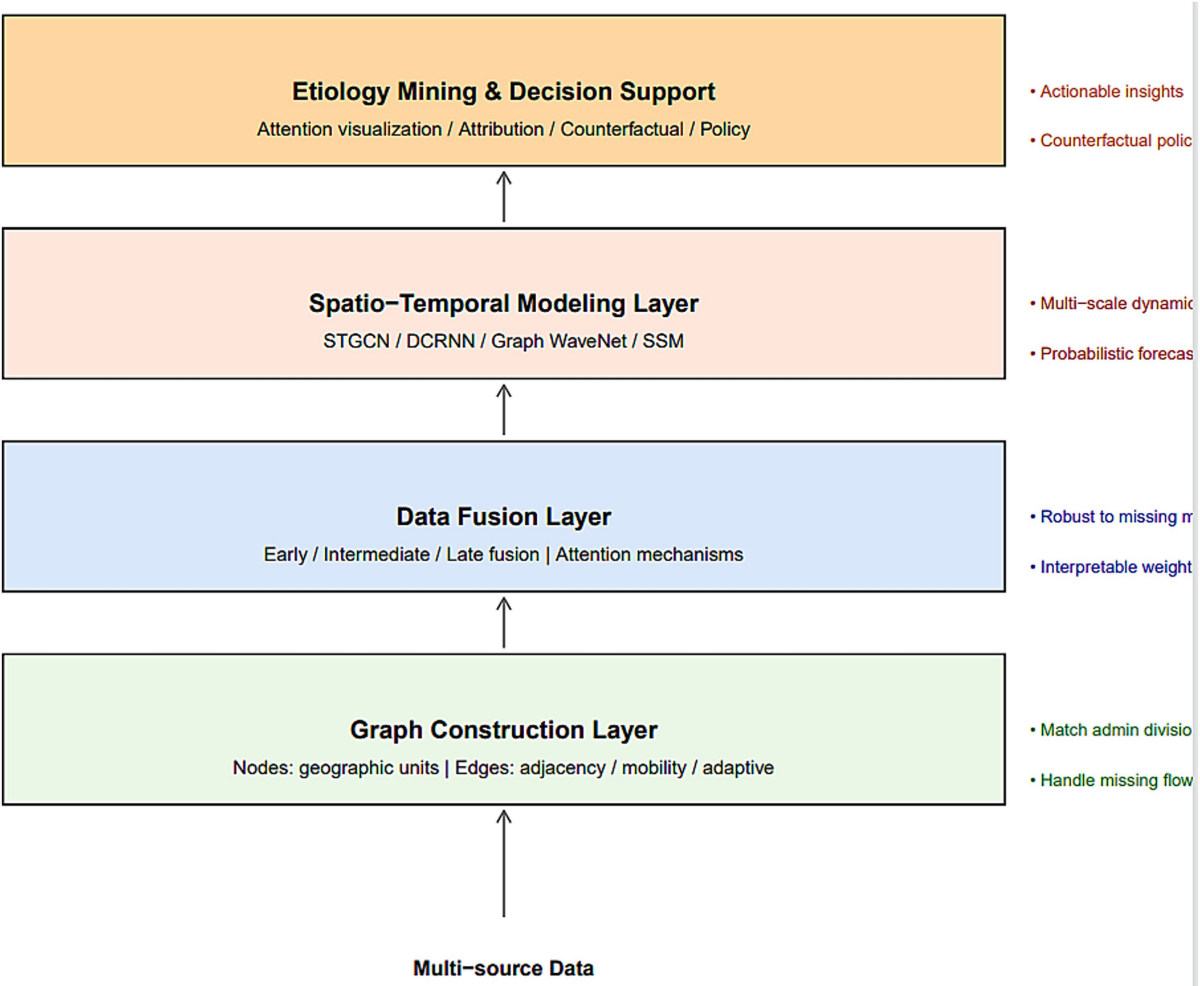
Four-layer methodological framework of ST-GNN for regional disease risk prediction and etiology mining.

Synthesizing existing research, this paper summarizes ST-GNN methods for regional disease risk prediction and etiology mining into a four-layer framework, supplemented with public health adaptability considerations for each layer. A methodological framework comprising four core layers is constructed (Figure 2).

### 3.1 Graph construction layer

#### 3.1.1 Technical pathway

Define nodes and edges. Nodes are typically geographical units (e.g., provinces, cities, districts/counties). Edges can be constructed based on the following relationships: (1) Geographical adjacency: based on administrative divisions; (2) Commuting flows: dynamic connections based on population mobility data; (3) Economic ties: similarity based on GDP, trade flows; (4) Dynamic adaptive graphs: data-driven learnable adjacency matrices.

#### 3.1.2 Public health adaptability

Graph structure design should align with the administrative divisions used in public health surveillance (e.g., districts/counties, cities) to directly support regionalized public health interventions. For example, a graph structure based on county-level geographical units enables prediction results to be directly used for delineating high-risk areas and allocating prevention and control resources. Furthermore, population mobility graphs need to consider the spatio-temporal granularity of mobility data—weekly commuting data are suitable for chronic transmission diseases, while daily mobility data are more valuable for emerging acute infectious diseases. In low- and middle-income regions where detailed mobility data may be lacking, alternative graph structures based on geographical adjacency or economic similarity should be designed to ensure the method’s generalizability.

### 3.2 Data fusion layer

#### 3.2.1 Technical pathway

Integrate multi-source heterogeneous data to generate high-dimensional node representations through the following strategies: (1) Concatenation fusion: direct concatenation of multi-source features; (2) Attention fusion: dynamic weighting of different modalities via cross-modal attention mechanisms; (3) Tensor fusion: modeling high-order interactions between modalities based on outer product operations; (4) Contrastive learning fusion: learning shared representations through contrastive loss between modalities.

#### 3.2.2 Public health adaptability

Multi-source data fusion must account for disparities in the availability of public health data. High-income regions may have access to detailed mobile phone signaling mobility data, while low- and middle-income regions may only have census data. Fusion strategies need to be robust and capable of handling data-missing scenarios—for example, when mobility data are absent, the model could degrade to relying solely on geographical adjacency graphs; when meteorological stations are sparse, spatial interpolation modules should be designed. Moreover, fusion results should remain interpretable, enabling public health personnel to understand the contribution weights of different modalities to the prediction.

### 3.3 Spatio-temporal modeling layer

#### 3.3.1 Technical pathway

The core prediction module models spatial and temporal dependencies in parallel or sequentially. Common architectures include: (1) STGCN: alternation of temporal convolution and graph convolution; (2) DCRNN: combination of diffusion convolution and gated recurrent units; (3) Graph WaveNet: dilated causal convolution with adaptive adjacency matrices; (4) Spatio-temporal attention networks: self-attention mechanisms based on Transformer; (5) State Space Models (SSM): structured state spaces capturing long-range dependencies.

#### 3.3.2 Public health adaptability

Spatio-temporal modeling should match the temporal scale of disease transmission. Acute infectious diseases (e.g., influenza) require capturing daily or weekly dynamics, while chronic diseases (e.g., diabetes) may involve monthly or quarterly changes. Models should possess multi-scale modeling capabilities—identifying short-term outbreak signals while grasping long-term trends. Furthermore, confidence intervals of predictions are crucial for public health decision-making; models should provide probabilistic forecasts rather than point estimates to support graded risk warnings.

### 3.4 Etiology mining layer

#### 3.4.1 Technical pathway

Reveal key factors and transmission paths influencing disease risk through the following methods: (1) Attention visualization: analyzing spatial/temporal attention weights; (2) Attribution analysis: calculating feature importance based on gradients, Shapley values, integrated gradients, etc.; (3) Counterfactual inference: simulating disease trajectories under intervention measures; (4) Causal structure learning: discovering causal relationships between variables from data.

#### 3.4.2 Public health adaptability

Results of etiology mining need to be translated into language understandable to public health personnel. For example, spatial attention weights can identify source regions contributing to “imported risk”; temporal attention can reveal critical time windows during the “incubation-outbreak” period; feature attribution can quantify the relative contributions of meteorological factors versus population mobility. These insights can directly support intervention strategy formulation—if population mobility is the main driver, transport quarantine should be strengthened; if meteorological factors dominate, vector control should be initiated early.

## 4 Disease risk prediction: from single-disease to multi-disease collaboration

### 4.1 ST-GNN applications in single-disease prediction

Single-disease prediction represents the most mature application scenario of ST-GNNs in epidemiology. For example, a study transferred the STGCN model to dengue fever prediction, integrating meteorological, socioeconomic, and environmental variables, achieving superior performance over random forest in nine American countries (R^2^ 0.78–0.98) (2). The key contribution of this study was demonstrating that ST-GNN architectures originating from urban computing can effectively capture the spatio-temporal heterogeneity of disease transmission. Model outputs can directly support public health departments in delineating high-risk areas and allocating prevention and control resources—for instance, before the dengue season, predictions can guide targeted mosquito breeding site elimination in high-risk areas, enabling precise intervention.

A more methodologically innovative approach is the Causality-Aware Deep Learning (CADL) framework proposed by Dang and Uyen (3), which integrates directed acyclic graph discovery, hybrid graph neural networks, Transformers, and structural causal models into a unified architecture for forecasting climate-sensitive infectious diseases (e.g., dengue). In synthetic experiments, CADL achieved a 26.8% reduction in mean absolute error compared to LSTM, Transformer, and GNN baselines, while also enabling counterfactual predictions under Representative Concentration Pathways (RCP) 4.5 and 8.5 climate scenarios, providing scenario-based risk quantification for policymakers.

For COVID-19 prediction, the STEP framework encoded inter-city mobility patterns as a graph structure, extracting implicit transmission features through multi-head attention mechanisms. Cola-GNN, designed for influenza forecasting, achieved long-term predictions via cross-regional attention. CSTGNN, proposed by Han et al. (4), combined physics-based SIR modeling with learnable spatio-temporal GCN embeddings, delivering both accurate predictions and interpretable epidemiological parameters (e.g., reproduction number R0 and adaptive contact matrices).

### 4.2 Multi-disease collaborative forecasting

The complex situation of concurrent epidemics of multiple diseases (e.g., influenza and RSV, hand-foot-mouth disease and dengue overlapping in time and space) places higher demands on prediction models. Liu and Cao (5) recently proposed a dynamic spatio-temporal graph attention network (ST-GAT) that, for the first time, simultaneously forecasts influenza, hand-foot-mouth disease, dengue, and RSV within a unified framework. The key innovations of this model are: (1) constructing a dynamic multi-relational graph that fuses both geographical adjacency and population flow relationships; (2) introducing a distribution-aware negative binomial decoder to address overdispersion in count data; (3) achieving cross-disease generalization improvement with a 34% reduction in MAE.

The advantage of multi-disease collaborative forecasting lies in the fact that different diseases may share similar transmission vectors or environmental drivers. Joint modeling helps extract common patterns and improves prediction performance in data-sparse regions. From a public health perspective, multi-disease collaborative forecasting can support the construction of integrated surveillance systems—simultaneously monitoring outbreak risks of multiple diseases within the same prediction platform, enhancing the overall early warning efficiency of public health systems.

### 4.3 Dynamic prediction in intensive care

The application of ST-GNNs extends beyond regional disease prediction to the individual level (e.g., intensive care), where they have also shown promise. The Graph-spa framework proposed by Yadav and Subbian (6) is a dynamic spatio-temporal graph neural network for the early prediction of acute respiratory distress syndrome (ARDS). Its core innovations include: (1) dynamically updating the adjacency structure to capture evolving interactions between clinical variables; (2) integrating temporal convolutional layers with ST-GNN to capture both local and non-local temporal dependencies; (3) combining mask-based interpretability methods to generate feature-time attribution scores, subsequently identifying sustained feature activation patterns during the 12 h preceding ARDS onset through co-occurrence analysis. On three datasets—HiRID, MIMIC-IV, and eICU—Graph-spa outperformed GRU, LSTM, TCN, Transformer, and STGNN baselines in terms of AUC, F1 score, and MCC.

### 4.4 Long-term forecasting and outbreak detection

In practical applications, public health decision-making requires both short-term warnings (1–4 weeks ahead) and long-term trend projections (quarterly or even yearly). Existing ST-GNNs excel in short-term prediction (especially capturing early transmission patterns) but face challenges in long-term extrapolation. A report from EPFL (2) noted that deep state space models (SSMs), by structuring latent spaces, can efficiently capture long-range dependencies, offering new avenues for long-term forecasting. Combining neural ordinary differential equations (Neural ODEs) with transmission graphs to build continuous-time dynamic models (e.g., the EARTH framework) enhances long-term prediction while maintaining interpretability.

Outbreak detection is another important direction. Studies have shown that ST-GNNs can identify anomalous signals within the early stages (1–2 weeks) of a surge in cases, gaining critical time windows for intervention measures. This capability aligns with the early warning requirements of the International Health Regulations (2005), which call for alerts as early as possible during the initial phase of rapid case increase to support timely public health emergency response.

## 5 Etiology mining: from prediction to understanding

If prediction answers the “what” and “when,” etiology mining answers the “why”—this is the core value of explainable artificial intelligence (XAI) and causal inference in public health.

### 5.1 Identification of spatial transmission paths

The graph attention mechanism in ST-GNNs inherently provides spatial attribution capabilities. By learning attention weights between nodes, the model can identify source regions that contribute most to the risk in a target area, thereby revealing disease transmission paths. Liu and Cao (5) showed that the spatial attention weights learned by ST-GAT were highly correlated with actual commuting flows, demonstrating that the model captures transmission patterns consistent with epidemiological intuition. Similarly, cross-regional attention in Cola-GNN can identify “hub cities” for influenza spread. From a public health perspective, these insights can support source control of “imported risk”—for example, if a city’s influenza risk primarily originates from a specific commuting hub, enhanced temperature screening and health education can be implemented at that hub.

Hancox’s doctoral thesis (7) systematically explored interpretability methods for temporal graph CNNs, comparing four explanation methods based on gradients and feature maps, providing visual insights for clinical decision-making. The study noted that while clinicians found these visualizations valuable, further simplification is needed to support real-world clinical decisions.

### 5.2 Temporal pattern attribution

Beyond the spatial dimension, models must answer: which historical time points are most critical for the current prediction? Temporal attention mechanisms play a role here. Studies have shown that ST-GAT (5) focuses its temporal attention on lag periods of 1–4 weeks, closely matching the incubation and transmission cycles of diseases like dengue. This temporal attribution lends biological plausibility to model predictions, enhancing decision-maker trust in the model.

The mask-based attribution method in Graph-spa (6) goes a step further, not only identifying critical time points but also discovering sustained feature activation patterns through co-occurrence analysis—for example, abnormal serum potassium levels combined with a declining Glasgow Coma Scale score jointly constituted an early risk feature combination for ARDS. Identifying such feature combinations can inform the development of clinical early warning rules.

### 5.3 Multi-factor interaction analysis

Disease transmission often results from complex interactions among multiple factors. For instance, temperature and precipitation directly affect mosquito density while also indirectly altering contact patterns by influencing human activity. Traditional regression models struggle to capture such interaction effects, whereas ST-GNNs can implicitly model these relationships through nonlinear transformations and multi-layer networks.

The CADL framework (3) introduces causal structure learning into deep learning, automatically identifying causal relationships (rather than mere correlations) between variables through directed acyclic graph discovery. This method can distinguish direct from indirect effects of meteorological factors, providing causal evidence for intervention design—if the effect of a factor is entirely mediated by population mobility, interventions should focus on mobility rather than directly controlling that factor. Additionally, post-hoc explanation methods such as Shapley values and integrated gradients are widely used for quantitative analysis of input feature importance.

### 5.4 Counterfactual inference and policy evaluation

A higher level of etiology mining is counterfactual inference: how would epidemic trajectories change if different interventions (e.g., travel restrictions, vaccination) were implemented? This is a core contribution of the CADL framework (3)—conducting counterfactual predictions under different climate change scenarios (RCP 4.5 and RCP 8.5) to support adaptive public health strategies. The ST-GAT study (5) embedded the prediction model into a multi-objective optimization engine to evaluate the cost-effectiveness of vaccine prioritization strategies, mobility restriction strategies, and their combinations. Results showed that vaccine prioritization performed best in terms of cost-effectiveness and equity. This direction marks the evolution of ST-GNNs from “prediction tools” to “decision support tools,” enabling public health decision-makers to make evidence-based choices among multiple intervention options.

## 6 Current challenges: technical bottlenecks and public health implementation constraints

Despite the great potential of ST-GNNs in disease risk prediction and etiology mining, the field faces systematic challenges. This section not only outlines technical bottlenecks but also supplements them with implementation constraints from public health practice. This section systematically reviews the five major technical challenges and their associated public health implementation constraints (Table 1).

**Table 1:** Correspondence between technical challenges and public health implementation constraints.

Technical challenge	Public health implementation constraint	Potential consequences
Dynamic graph structure modeling	Real-time responsiveness for emergency response	Model update lag leads to prediction inaccuracies, missing intervention windows
Cross-modal heterogeneous fusion	Data scarcity in low- and middle-income regions	Data gap exacerbates health inequalities
Trade-off between prediction and interpretability	Diverse explanation needs of multiple stakeholders	Decision-makers find it hard to trust and adopt model results
Out-of-distribution generalization	Cross-regional transfer application	Hinders global public health technology transfer
Privacy-preserving federated learning	Differences in data regulations, cross-agency coordination	Data sharing barriers, limited model performance

### 6.1 Dynamic graph structure modeling

Technical challenge: Most existing studies rely on static graphs (fixed adjacency matrices), but real-world disease transmission networks are dynamic: population mobility varies with seasons and policies, new transportation links alter regional connectivity, and public health interventions temporarily disrupt transmission paths. How to incorporate dynamic graph structures (i.e., graphs that change over time) into models remains an open problem. Frameworks like ROLAND for dynamic graph learning provide initial ideas, and Graph-spa (6) demonstrates dynamic graph potential by updating adjacency matrices, but systematic applications in epidemiological scenarios are still lacking.

Public health implementation constraint: Public health interventions (e.g., lockdowns, traffic restrictions) dynamically alter disease transmission networks, and the speed of model updates must match the real-time requirements of public health emergency response, as emphasized in Section 3.1—graph structure design must align with the timeliness of public health emergency response. For example, during the early phase of an outbreak, intervention policies may be adjusted daily, requiring models to quickly respond to changes in graph structure. If model updates lag by several days, predictions may severely deviate from actual transmission patterns, missing intervention windows—suppose a city implements traffic controls the day after the first case is detected, but a static-graph model continues to predict future risk based on the original mobility patterns; it would overestimate the outbreak size under no intervention, misleading resource allocation.

### 6.2 Cross-modal heterogeneous fusion

Technical challenge: Although multi-source data are abundant, significant heterogeneity exists between modalities: meteorological data are high-frequency continuous values, socioeconomic data are low-frequency discrete values, and textual data (e.g., news reports, policy documents) are unstructured information. Current mainstream practices convert unstructured data into structured features, a process that may lead to information loss. The three-layer architecture of “digital omics–digital biomarkers–digital phenotypes” proposed by Xue (8) provides a theoretical framework for multimodal cohort design, but its computational complexity and interpretability require further validation. Recent studies have begun exploring deep integration of vision-language models with graph neural networks (9), but clinical applications remain in early stages.

Public health implementation constraint: Multi-source data fusion must consider disparities in the availability of public health data—low- and middle-income regions may lack mobile phone signaling data, fine-grained meteorological station data, and may only have census data and manually reported cases. Fusion strategies need “graceful degradation” capabilities: when fine-grained data are missing, the model should still produce reasonable predictions based on coarse-grained data, avoiding exacerbation of health inequalities due to data gaps. For example, in sub-Saharan Africa, if mobility data are unavailable, geographical adjacency graphs combined with nighttime light data (as a proxy for economic activity) could still provide reference-worthy predictions.

### 6.3 Trade-off between prediction and interpretability

#### 6.3.1 Technical challenge

High prediction accuracy often relies on complex deep networks, which in turn often sacrifice interpretability. Although attention mechanisms provide some explanation, there is debate in academia about whether they truly reflect the model’s decision-making basis. Studies have shown that attention weights may be inconsistent with gradient-based attributions. Hancox (7) indicated that clinicians require more simplified visual explanations to support real-world decisions. How to maintain prediction accuracy while providing faithful explanations remains a research hotspot.

#### 6.3.2 Public health implementation constraint

Public health decision-making involves multiple stakeholders—policymakers, frontline health workers, and the public—each with different needs for interpretability. Policymakers focus on “which factors are actionable,” frontline workers care about “where are the high-risk areas,” and the public wants to know “how to protect themselves personally.” Models need to provide multi-level, customizable explanation outputs to meet the decision-making needs of different roles. For instance, policymakers could be presented with feature importance rankings and counterfactual simulation results, while the public could receive simplified risk level maps and protective recommendations.

### 6.4 Out-of-distribution generalization

#### 6.4.1 Technical challenge

ST-GNNs perform excellently on time periods and regions within the training distribution, but performance often degrades significantly when faced with emerging diseases (no historical data), drastic changes in transmission patterns due to policy interventions, or when transferring models to different geographical regions. This out-of-distribution (OOD) generalization problem is a key bottleneck for transitioning ST-GNNs from academic research to practical application. Causal representation learning and structured state space models are considered potential solutions. EPFL’s report (2) noted that state space models, through structured latent spaces, can generalize better to OOD samples.

#### 6.4.2 Public health implementation constraint

Low- and middle-income regions have sparse disease data and transmission patterns that differ significantly from high-income regions, making cross-regional generalization of ST-GNNs a critical technological bottleneck for global health equity. For example, an influenza prediction model trained on European/US data, directly transferred to Africa, may fail due to differences in climate, population structure, and healthcare conditions. Lightweight, transferable model adaptation methods such as transfer learning and meta-learning need to be developed to support global public health technology transfer. A recent WHO report (10) also emphasizes that AI models must be adapted to different resource settings to avoid technological inequality arising from a “one-size-fits-all” approach.

### 6.5 Privacy-preserving federated learning

#### 6.5.1 Technical challenge

Multi-source data are often distributed across different institutions (CDC, meteorological bureau, telecom operators) and cannot be easily centralized due to privacy and data security regulations. Federated learning offers a technical pathway—models are trained locally, and only gradient updates are aggregated. However, adapting ST-GNNs to federated learning presents challenges: sharing graph structures across institutions may leak topological information, and statistical distribution differences in heterogeneous data affect model convergence. Chen et al. (11) systematically reviewed federated learning applications in infectious disease surveillance, identifying current technical limitations.

Public health implementation constraint: Sharing public health data among CDCs, meteorological bureaus, and telecom operators faces not only technical challenges (e.g., topological information leakage) but also constraints from varying data management regulations across regions. For example, the EU’s General Data Protection Regulation (GDPR) and China’s Data Security Law impose different requirements on cross-institutional data sharing. Solutions require coordinated technological and policy efforts—technically, designing privacy-preserving graph federated learning protocols (e.g., differential privacy, secure multi-party computation); and at the policy level, promoting the establishment of cross-institutional data sharing standards and governance frameworks. Gostin and Friedman (12) point out that achieving global health equity requires simultaneously addressing technological capacity and legal harmonization.

## 7 Future directions: public health-oriented technological development

### 7.1 Integration of foundation models and ST-GNNs

Large language models and vision foundation models have demonstrated powerful general-purpose capabilities in multimodal understanding tasks. Shen et al. (13) noted that large AI models offer significant advantages in large-scale data processing, multimodal data fusion, recognition of complex interaction patterns, and construction of high-precision prediction models, bringing new opportunities to epidemiological research. A foreseeable direction is to use pre-trained foundation models as “front-end encoders” for ST-GNNs to extract implicit knowledge from meteorological texts, medical literature, policy documents, etc., enhancing the semantic understanding capability of disease prediction. The multimodal cohort design framework based on AI language representation proposed by Xue (8) provides theoretical support for this direction.

#### 7.1.1 Public health orientation

Pre-trained foundation models need to be adapted to low-resource public health data settings (e.g., data-scarce regions in developing countries). Techniques such as transfer learning and few-shot learning should be employed to improve model adaptability across different public health scenarios. For example, a foundation model pre-trained on global data could be fine-tuned with a small amount of local data to rapidly build a prediction system suitable for low- and middle-income regions. Murray (14) noted in The Lancet Digital Health that this “pre-training + fine-tuning” paradigm is a key pathway to narrowing the global digital health divide.

### 7.2 Cognitive intelligence and knowledge augmentation

Current ST-GNNs are primarily data-driven and do not fully exploit domain knowledge from epidemiology. Incorporating transmission dynamics equations (e.g., SEIR models) as physics-informed losses for neural networks, or introducing prior knowledge of transmission pathways into graph structures, holds promise for maintaining reasonable and interpretable predictions even when data are sparse. CSTGNN (4) has made initial explorations in this direction by combining SIR modeling with GCN embeddings. Future work could further incorporate causal graphs as prior knowledge into models, achieving “data-driven + knowledge-guided” hybrid intelligence.

#### 7.2.1 Public health orientation

Knowledge augmentation needs to incorporate localized epidemiological parameters—incubation periods, infectious periods, and reproduction numbers differ across diseases, and transmission parameters for the same disease may vary across populations. Models should possess parameter adaptation capabilities, dynamically adjusting knowledge constraints based on real-time data. For example, during a measles outbreak, the model could automatically load measles-specific transmission dynamics parameters and adjust them using local vaccination coverage data to improve prediction accuracy.

### 7.3 Causal inference and counterfactual analysis

The CADL framework (3) has demonstrated that combining causal structure learning, counterfactual simulation, and deep learning can simultaneously improve prediction accuracy and interpretability. Future directions include: (1) moving from synthetic data to real-world data validation; (2) extending causal discovery from static graphs to spatio-temporal dynamic causal graphs; (3) establishing evaluation standards for counterfactual predictions. This direction could elevate ST-GNNs from “prediction models” to “causal models,” providing a more solid scientific basis for public health interventions.

#### 7.3.1 Public health orientation

Counterfactual analysis should be integrated with cost-effectiveness evaluation of public health interventions. For example, simulating the epidemiological effects and economic costs of different vaccine allocation strategies can support decision-makers in making optimal choices under resource constraints. Furthermore, validation frameworks for counterfactual predictions need to be established—since the real world cannot simultaneously observe both intervention and non-intervention states, quasi-experimental methods (e.g., regression discontinuity, difference-in-differences) should be designed to assess the accuracy of counterfactual predictions. Wang et al. (15) have made initial explorations in this area.

### 7.4 Closed-loop optimization from prediction to decision

Prediction itself is not the end; intervention decision-making is. Combining ST-GNNs with reinforcement learning and multi-objective optimization to build a “prediction–intervention–evaluation” closed-loop system—where the model can not only predict “what will happen if nothing is done” but also recommend “what should be done” and evaluate the consequences—is a crucial step toward intelligent public health decision-making. The ST-GAT study (5) has preliminarily demonstrated the feasibility of this direction.

#### 7.4.1 Public health orientation

When constructing the “prediction–intervention–evaluation” closed loop, participation from public health policymakers and frontline health workers should be ensured to guarantee that the intervention strategies recommended by the model align with local public health resource realities and social acceptability. Specifically, future research should design quantifiable feedback indicators: (1) Intervention cost: economic expenditure for each intervention measure; (2) Health benefit: cases averted, deaths averted, quality-adjusted life years (QALYs); (3) Equity indicators: disparities in benefits across income groups, urban/rural areas. This requires interdisciplinary collaboration with health economics and policy evaluation to establish a cross-disciplinary evaluation framework. For example, the objective function of reinforcement learning could be designed to maximize the weighted sum of health benefits and equity, subject to cost constraints.

### 7.5 Fairness and algorithm auditing

Equity in resource allocation is a core ethical consideration in public health decision-making. Future research needs to address: Are there systematic differences in ST-GNN prediction errors across regions or populations? Does the model produce less accurate predictions for already resource-scarce areas? Incorporating fairness constraints into model design and evaluation is an essential requirement for responsible AI. Ethical challenges in multimodal AI fusion have been noted in reviews (16) and need to be concretely implemented in algorithmic designs. Finally, algorithm auditing should be integrated into the regulatory process for public health technology applications. We recommend that model evaluation standards mandatorily include performance audit reports for vulnerable populations and regions, ensuring that models pass fairness tests before deployment. Post-deployment monitoring of fairness indicators should be continuous, enabling timely detection and correction of emergent biases. As The Lancet Public Health has emphasized, health equity must be a core design principle of digital public health technologies, not an afterthought added after model development (19). Furthermore, systematic auditing frameworks must account for disparities in healthcare resource allocation to prevent AI systems from inadvertently widening existing inequalities (20).

#### 7.5.1 Public health orientation

A public health fairness evaluation framework for ST-GNN models needs to be established, including: (1) differences in prediction errors across income-level regions; (2) differences in prediction errors across age-structure populations; (3) mechanisms to ensure model performance in resource-scarce areas. Future model evaluation standards should mandatorily include performance audit reports for vulnerable populations/regions to ensure models pass fairness tests before deployment. Zhang et al. (17) proposed a systematic fairness assessment framework including error decomposition, redistribution analysis, and counterfactual fairness testing. Furthermore, algorithm auditing should be integrated into the regulatory process for public health technology applications, ensuring continuous monitoring of fairness indicators after deployment and timely detection and correction of potential biases. The Lancet Public Health (18) calls for health equity to be a core design principle of digital public health technologies.

## 8 Conclusion

This review systematically examined research progress on spatio-temporal graph neural networks and multi-source data fusion in regional disease risk prediction and etiology mining. From a methodological perspective, ST-GNNs integrate spatial dependencies, temporal dynamics, and multimodal features through a unified graph learning framework, surpassing traditional models in prediction accuracy, and revealing key drivers of disease transmission through attention mechanisms, attribution analysis, and causal inference. From an application perspective, the paradigm has expanded from single-disease prediction to multi-disease collaborative forecasting, dynamic prediction in intensive care, long-term extrapolation, and intervention optimization.

However, challenges remain in dynamic graph structures, out-of-distribution generalization, privacy protection, and other areas, requiring coordinated efforts from academia and industry. Looking ahead, when ST-GNNs can deeply integrate with foundation models, incorporate causal inference capabilities, stably provide accurate, interpretable, and fair predictions, and be deeply embedded into public health decision-making processes, we can truly achieve the leap from “seeing the epidemic” to “steering the epidemic”.

In the future, the deep integration of ST-GNNs and public health will not only enable the leap from “seeing the epidemic” to “steering the epidemic” but also provide technological support for the modernization of global public health governance systems, helping to narrow the technological gap between different regions and advancing the global goal of health equity.
